# Purinergic Enhancement of Anti-Leishmanial Effector Functions of Neutrophil Granulocytes

**DOI:** 10.3389/fimmu.2021.747049

**Published:** 2021-10-18

**Authors:** Sonja Möller, Tamás Laskay

**Affiliations:** Department of Infectious Diseases and Microbiology, University of Lübeck, Lübeck, Germany

**Keywords:** *Leishmania donovani*, ATP, UTP, purinergic, intracellular killing, neutrophils

## Abstract

Although macrophages are considered for host cells for the multiplication of *Leishmania*, recent studies indicate the important role of neutrophil granulocytes as host cells for these intracellular parasites. Neutrophils have been shown to be massively and rapidly recruited to the site of *Leishmania* infection where they represent the first cells to encounter the parasites. Exposure to ATP and UTP have been shown to enhance anti-*Leishmania* activity of macrophages and intralesional injection of UTP led to strongly reduced parasite load *in vivo*. Since the *in vivo* anti-leishmanial effect of extracellular UTP correlated with enhanced neutrophil recruitment and enhanced ROS production at the site of *Leishmania* infection we hypothesized that exposure to extracellular nucleotides can directly enhance the killing of *Leishmania* by neutrophils. Since purinergic signaling is an essential mechanism of neutrophil activation the aim of the present study was to assess whether purinergic exposure results in the activation of anti-leishmanial neutrophil functions and, therefore, represent an essential component of enhanced anti-leishmanial defense in leishmaniasis. We could show that exposure to ATP and UTP led to activation and enhanced CD11b expression of primary human neutrophils *in vitro*. *Leishmania*-induced ROS production was strongly enhanced by extracellular ATP and UTP. Importantly, exposure to ATP and UTP resulted in enhanced killing of *Leishmania donovani* by neutrophils. In addition, ATP strongly enhanced the secretion of IL-8 and IL-1β by *Leishmania*-exposed neutrophils. Our results suggest that signaling *via* the P2 receptor and phosphorylation of Erk1/2, Akt and p38 are involved in the purinergic enhancement of anti-leishmanial functions of neutrophils.

## Introduction

Leishmaniases comprise vector-borne parasitic diseases with multiple clinical manifestations caused by the unicellular protozoon parasites of the genus *Lesihmania*. Visceral leishmaniasis (VL) is the most serious form of leishmaniasis and has an incidence of estimated 400 000 cases per year (source: Centers for Disease Control and Prevention). In the mammalian host *Leishmania* parasites are obligatory intracellular pathogens. Since *Leishmania* parasites cannot actively penetrate and invade host cells, *Leishmania* depend on the phagocytic activity for their entry into their host cells. Although classically macrophages are considered for host cells for the multiplication of *Leishmania*, recent studies indicate the important role of neutrophil granulocytes as host cells for these intracellular parasites. Neutrophils have been shown to be massively and rapidly recruited to the site of *Leishmania* infection where they represent the first cells to encounter the parasites (1, 2). Neutrophils can play both protective as well as disease promoting roles in leishmaniasis. On the one hand neutrophils participate in the elimination of parasites through the production of reactive oxygen species (ROS), the release of azurophilic granules that contain antimicrobial proteins such as neutrophil elastase (NE) and myeloperoxidase (MPO) (3, 4). In addition, neutrophil extracellular traps (NETs) have been described to kill some *Leishmania* spp (5). On the other hand, neutrophils can also play a detrimental role in leishmaniasis (6, 7). Neutrophils can provide a transient intracellular niche for the intracellular pathogen and function as “Trojan horses” to transfer the parasites to macrophages (8, 9).

Adenosine triphosphate (ATP) and uridine triphosphate (UTP) are intracellular nucleotides that are essential for nucleic acid synthesis as well as source of energy. However, extracellular ATP and UTP represent damage associated molecular patterns (DAMPs) that modulate inflammatory and immune responses against infectious agents (10). The level of extracellular ATP can reach hundreds of micromolar concentration (11). Extracellular ATP and UTP act on leukocytes upon binding to surface P2 receptors. P2 receptors include the P2X and P2Y receptors. P2X receptors function as ligand-gated ion channels. ATP is a native agonist for all seven P2X receptors (12, 13). P2Y receptors are G-protein coupled receptors (GPCR). Preferred agonists of P2Y receptors are the purines ATP and ADP, the pyrimidines UTP, UDP and UDP-sugars (12–14). ATP can bind to all eight P2Y receptors except to the P2Y6 receptor. UTP is a native agonist of P2Y2, P2Y4 and P2Y11 (12, 13).

Exposure to ATP and UTP have been shown to enhance anti-*Leishmania* activity of macrophages (15). Parasite load in *L. amazonensis* infected macrophages was reduced *in vitro* after treatment with ATP (16). In an experimental model of *Leishmania* infection intralesional injection of UTP led to strongly reduced parasite load *in vivo* (17). Since the *in vivo* anti-leishmanial effect of extracellular UTP correlated with enhanced neutrophil recruitment and enhanced ROS production at the site of *Leishmania* infection (17) we hypothesized that exposure to extracellular nucleotides can directly enhance the killing of *Leishmania* by neutrophils and this effect play a substantial role in the anti-leishmanial effect of extracellular nucleotide treatment in *Leishmania* infection.

Neutrophils express several P2X and P2Y receptors and signaling *via* purinergic receptors has been shown to represent a fundamental mechanism of neutrophils activation (18). Purinergic signaling control PMN chemotaxis, modulate life span, and activate neutrophil functions such as phagocytosis and ROS release and were reported to be required for activation of PMN by a wide variety of stimuli (18, 19). Since purinergic signaling is an essential mechanism of neutrophil activation (20) the aim of the present study was to assess whether purinergic exposure results in the activation of anti-leishmanial neutrophils functions and, therefore, represent an essential component of enhanced anti-leishmanial defense in leishmaniasis. We could show that exposure to ATP and UTP strongly enhance anti-leishmanial functions of primary human neutrophils *in vitro*. Our results suggest that signaling *via* the P2 receptor and phosphorylation of Erk1/2, Akt and p38 are involved in the purinergic enhancement of anti-leishmanial functions of neutrophils.

## Materials and Methods

### Isolation of Human Neutrophil Granulocytes

Primary human neutrophils were isolated from peripheral blood of healthy volunteers by Histopaque and Percoll gradient centrifugation as described elsewhere (21). The blood collection was conducted with the understanding and written consent of each participant and was approved by the ethical committee of the Medical Faculty of the University of Lübeck (18-187). Blood was layered on a two-layer density gradient consisting of lymphocyte separation medium 1077 (upper layer, PAA, Pasching, Austria) and Histopaque^®^ 1119 (bottom layer, Sigma-Aldrich, Deisenhofen, Germany) and centrifuged for 5 min at 300 × g followed by 20 min at 800 × g. Cells from the upper layer consisting mainly of lymphocytes and monocytes were discarded. The granulocyte-rich lower layer was collected leaving the erythrocyte pellet at the bottom of the tube. Granulocytes were washed once in PBS, resuspended in complete medium (RPMI 1640 medium, Sigma) supplemented with 50 µM 2-mercaptoethanol, 2 mM L-glutamine, 10 mM HEPES (all from Biochrom, Berlin, Germany) and 10% fetal calf serum (FCS, Gibco, Karlsruhe, Germany) and further fractionated on a discontinuous Percoll^®^ (Amersham Biosciences, Uppsala, Sweden) gradient consisting of layers with densities of 1.105 g/ml (85%), 1.100 g/ml (80%), 1.093 g/ml (75%), 1.087 g/ml (70%), and 1.081 g/ml (65%). After centrifugation for 20 min at 800 × g, the interface between the 80 and 70% Percoll^®^ layers was collected, washed once in PBS and resuspended in complete medium. All procedures were conducted at room temperature. The cell preparations contained > 99.9% granulocytes as determined by morphological examination of > 1,000 cells on Giemsa stained cytocentrifuge (Shandon, Pittsburgh, PA) slides. Cell viability was > 99%, as determined by trypan blue exclusion.

### Flow Cytometry Analysis of Cell Surface Expression of Activation Markers

Neutrophils (500.000 cells/sample) were resuspended in FACS buffer [1x PBS (Thermo Fisher) supplemented with 1% bovine serum albumin and 1% human serum] in a V-bottom plate. After washing once with FACS buffer the cells were stained with FITC-conjugated mAb to human CD62L (clone DREG-56, IgG1, BD Biosciences) and PE-conjugated mAb to human CD11b (clone 2LPM19c, IgG1, Dako, Waldbronn, Germany) for 30 min at 4°C protected from light. After two wash steps with FACS buffer at 4°C the cells were resuspended in FACS buffer and measured with a BD FACS Canto II (BD) flow cytometer.

### Assessment of Neutrophil Apoptosis and Viability

Apoptosis of neutrophils was determined by Annexin-V FLUOS binding as Annexin-V exhibits calcium-dependent binding to phosphatidylserine (PS) expressed in the outer leaflet of the cell membrane of apoptotic neutrophils. Assessment of membrane integrity and identification of necrotic cells was performed by counterstaining with propidium iodide (PI). For staining, 5×10^5^ cells/100 µl were transferred to a U-tube and 10 µl Annexin-V buffer (10 mM HEPES + 140 mM NaCl, pH 7.4), 1 µl 1 M CaCl_2_, 1 µl PI (Sigma-Aldrich) and 1 µl Annexin-V FLUOS (Sigma-Aldrich) were added. Samples were incubated 10 min at 4°C protected from light. Analysis was carried out by flow cytometry.

### *Leishmania donovani* Culture and Staining With CFSE

Virulent *L. donovani* promastigotes (strain MHOM/IN/82/Patna 1) were cultivated in Schneider’s Drosophila Medium with L-glutamine (Genaxxon, Ulm, Germany) supplemented with 10% FCS, 100 U/ml penicillin, 100 μg/ml streptomycin, and 2% sterile filtered human urine at 27°C in a humidified air atmosphere containing 5% CO_2_. For seeding of cultures, a parasite density of 1 × 10^6^ cells/ml was used. The parasite counting was conducted in a hemocytometer with a chamber depth of 0.02 mm (0.0025 mm^2^, depth 0.02 mm, VWR). The culture was considered to be in the stationary phase 72 h after seeding. Serial passaging was conducted until passage 10.

Stationary phase *L. donovani* promastigotes were labeled with CFSE (Cell Trace CFSE, Invitrogen, Thermo Fischer Scientific) by incubating the parasites in a volume of 1 ml at a concentration of 1x10^8^/ml with 5 µM CFSE in PBS for 15 min. with shaking. 500 µl complete medium was then added and incubated for another 5 min with shaking. Cell were then centrifuged at 1300 x g and resuspended in complete medium.

### *In Vitro* Infection of Neutrophils With *Leishmania donovani* Promastigotes

Neutrophils and *L. donovani* promastigotes were co-incubated in complete medium at a parasite-neutrophil ratio of 10:1 and a neutrophil density of 5 × 10^6^ cells/ml for 3 h at 37°C in a humidified air atmosphere containing 5% CO_2_. Subsequently, the infection rate was assessed on Giemsa-stained cytocentrifuged samples. The cells were washed six times in 1 × PBS at 400 × g for 10 min to remove extracellular *Leishmania*. Infected cells were again adjusted to 5 × 10^6^ cells/ml.

### ROS Assay

The luminol-based chemiluminescence assay was used to detect the sum of intra- and extracellular ROS. 4×10^5^ neutrophils (2x10^6^ cells/ml) were seeded per well in a 96-well LUMITRAC™ 600 plates (Greiner Bio-One, Frickenhausen Germany) and mixed with a final concentration of 60 µM luminol (Sigma-Aldrich). Neutrophils were exposed to ATP (500 µM), UTP (500 µM), or LPS (100 ng/ml) or fMLP (1 µM). ROS-dependent luminol chemiluminescence was assessed using an infinite 200 reader and the Tecan i-control 1.7 Software (Tecan, Crailsheim, Germany). ROS release was monitored for 2 h every 2 min at 37°C. For statistical analysis, the area under the curve (AUC) value of each sample was calculated.

To assess the effect of ATP and UTP on the ROS production of *Leishmania*-exposed neutrophils, neutrophils were co-incubated with *L. donovani* promastigotes in the presence or absence of ATP or UTP and the ROS production was assessed for 60 min by using the luminol based chemiluminescence assay.

### Assessment of Intracellular Ca^2+^-Concentration

Intracellular Ca^2+^-concentration was determined by using a flow cytometry-based method with the fluorescent Ca^2+^-sensitive indicator Fluo-4 AM as described (22). Briefly, neutrophils, 1x10^7^/ml, were labeled with 10 µM Fluo-4 AM (AAT Bioquest, Sunnyvale, CA) for 30 min in the dark. After washing with PBS cells were resuspended in complete medium at a concentration of 5x10^6^/ml. Neutrophils were infected with *Leishmania* by co-incubating neutrophils with *L. donovani* promastigotes at a neutrophil to *Leishmania* ratio of 1:10 for 2h at 37°C. ATP (500 µM) or UTP (500 µM) was added to the infected neutrophils. Fluorescence intensity was assessed for 3 min in FITC channel by using a BD FACS Canto II flow cytometer (BD). The stimuli were added at the time point 30 sec of the flow cytometry analysis. Fluo-4-labeled non-infected cells were used as negative controls, Fluo-4-labeled non-infected cells exposed to ionomycin (10 µM) were used as positive control.

### Western Blot Analysis

Neutrophils (5×10^6^ cells/ml) were exposed to ATP (500 µM), to *Leishmania donovani* promastigotes (*Leishmania* to neutrophil ratio 10:1) or a combination of ATP with *Leishmania donovani* promastigotes for 15 min at 37°C. In samples with the P2-antagonist PPADS (Tocris, Bio-Techne, Wiesbaden, Germany) the neutrophils were pretreated with the inhibitor (30 µM) for 15 min. Whole cell lysates were prepared as described (23). Western blot analysis was carried out by using antibodies against human phospho Akt (Thr308), phospho-p44/42 MAPK (ERK1/2, thr202/Tyr204), phospho p38 MAPK (Thr180/Tyr182) and beta-actin (all from Cell Signaling Technology) and probed with HRP-conjugated anti-rabbit IgG secondary antibody (New England Biolabs, USA). The signals were detected by using Immobilon Western Chemiluminescence HRP substrate (Millipore, USA) with a Fusion Fxt Chemiluminescence reader (Vilber Loumat, Germany). Signals of pAkt, pERK1/2, or pp38 were related to beta-actin signals on the same blots or of the same sample by using ImageJ software (NIH, USA). Data were then normalized by sum of all data point in a replicate as described (24).

### Phagocytosis Assays

#### Phagocytosis of Latex Microspheres

Neutrophils (5×10^5^ cells/100 μl) were preincubated for 30 min with ATP (500 µM), UTP (500 µM), LPS (100 ng/ml) or a combination of LPS with ATP or UTP. Subsequently, FluoSphere carboxylate-modified latex microspheres (Invitrogen™) with a diameter of 1 μm at a final concentration of 0.015% (v/v) were added and the co-culture was incubated for further 30 min. Cultures were placed on ice to stop phagocytosis and cells were washed to remove non-ingested beads. Phagocytosis was assessed by flow cytometry using a FACS Canto II flow cytometer (BD).

#### Phagocytosis of *L. donovani* Promastigotes

Neutrophils (5×10^5^ cells/100 μl) were preincubated for 30 min with ATP (500 µM), UTP (500 µM) or left untreated. Subsequently, neutrophils were co-incubated with CFSE-labeled *L. donovani* promastigotes in 200 µl complete medium in flat-bottom 96-well plate at a parasite-neutrophil ratio of 5:1 and a neutrophil density of 5 × 10^6^ cells/ml for 120 min at 37°C in a humidified air atmosphere containing 5% CO_2_. *L. donovani* promastigotes were labeled with Cell Trace CFSE (Invitrogen) according to the manufacturer’s protocol. Phagocytosis was assessed by flow cytometry using a BD FACS Canto II flow cytometer (BD). The infection rate was also checked microscopically on Giemsa-stained cytocentrifuged samples in order to control whether the CFSE-labeled promastigotes were inside the neutrophils rather than attached to the outer surface.

### Cytokine Determination

Neutrophils (1x10^6^ cells/100 µl) were exposed to ATP (500 µM), UTP (500 µM), *L. donovani* promastigotes or a combination of ATP and UTP with *L. donovani* promastigotes or medium alone for 18 h at 37°C, 5% CO_2_. Cultures were centrifuged at 300 x g for 10 minutes to separate cytokine-containing supernatants from cells. Detection of IL-8 and IL-1β in the supernatants was conducted by using Duo-Set ELISAs (R&D Systems) according to the manufacturer’s instructions.

### Assessment of Killing of *Leishmania* by Neutrophils

*Leishmania*-infected neutrophils were exposed to ATP (500 µM), UTP (500 µM) or LPS (100 ng/ml) as well as to the combination of LPS with ATP and UTP for 18 h at 37°C in a humidified air atmosphere containing 5% CO_2_. A limiting dilution culture assay was then used to detect viable *L. donovani* parasites in neutrophils and survival of *L. donovani* parasites as described previously (25). Briefly, serial 1.5-fold dilutions of *L. donovani*-infected neutrophil suspensions (5 × 10^6^ cells/ml) were plated in three replicates in 96-well flat-bottom microtiter plates containing Schneider’s Drosophila Medium. The plates were incubated at 27°C in humidified air atmosphere containing 5% CO_2_ for 7-10 days. The growth of *L. donovani* promastigotes was detected microscopically. The last dilution resulting in a growth of parasites in > 50% of the wells is given as a quantitative measure of the parasite load in the neutrophil cell suspension.

### Statistical Analysis

If not stated differently, the presented data were collected/generated from minimum of three independent experiments with neutrophils isolated from different blood donors. Statistical analysis was performed with the GraphPad Prism software 6 using the ordinary one-way ANOVA followed by Turkey’s multiple comparison. A p-value ≤0.05 was considered statistically significant.

## Results

### Effect of Extracellular ATP and UTP on CD62L Shedding and CD11b Expression on Neutrophil Granulocytes

**Figure 1:**
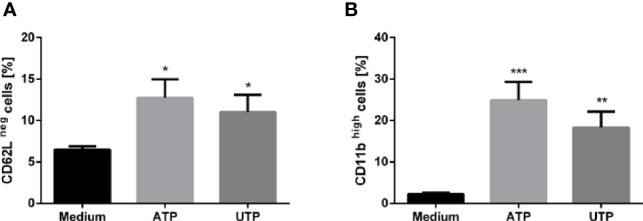
The effect of extracellular ATP and UTP on the cell surface expression of CD62L and CD11b on neutrophils. Primary human neutrophils were incubated for 30 in the presence of ATP (500 µM) or UTP (100 µM). The expression of CD62L **(A)** and CD11b **(B)** was assessed by using flow cytometry. Bar diagrams show % ± SD (n=3). *p < 0.05, **p < 0.01, ***p < 0.001.

Since activation of PMN leads to shedding of L-selectin (CD62L) from the cell surface (26), analysis of CD62L expression is a widely used method to distinguish CD62L^neg/low^ activated cells from CD62L^high^ non-activated neutrophils (27). To obtain information regarding the activation status of neutrophils upon exposure to ATP and UTP the expression of L-selectin (CD62L) was assessed *via* flow cytometry. As compared to untreated cells, a marked shedding of CD62L was observed in neutrophils upon a 30 min exposure to ATP and UTP (**Figure 1A**).

Granule exocytosis is an important function of activated neutrophils. During this process, the granule membrane incorporates into the cell membrane. Consequently, increasing expression of granule-membrane markers such as CD11b (28) can be observed on the cell surface of activated PMN. To investigate whether exposure to ATP and UTP affects granule exocytosis the cell surface expression of the degranulation marker integrin alpha M (CD11b) was assessed by using flow cytometry. The expression of CD11b was significantly enhanced on ATP- and UTP-treated neutrophils. (**Figure 1B**). These data indicate an activated state and granule exocytosis of neutrophils upon exposure to extracellular ATP and UTP.

### Effect of Extracellular ATP and UTP on the Production of ROS by Neutrophils

**Figure 2:**
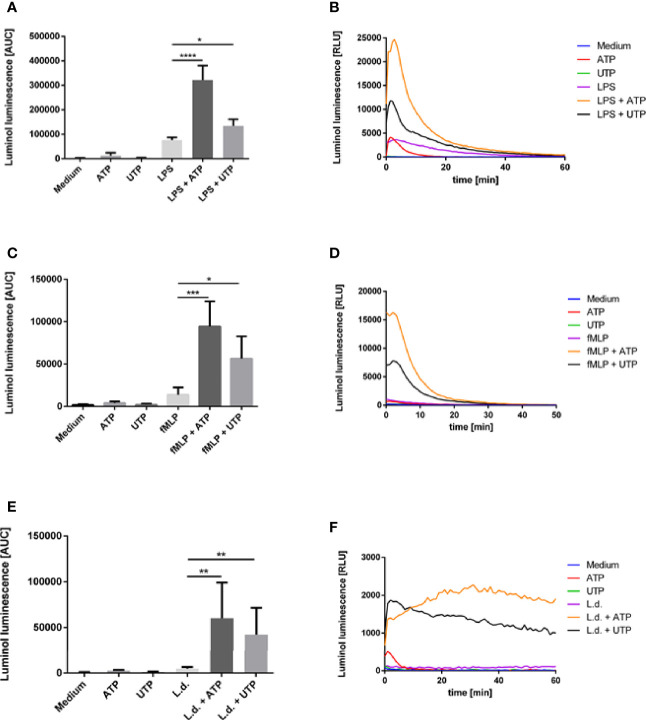
The effect of extracellular ATP and UTP on the ROS production by neutrophils. Neutrophils were treated with **(A, B)** ATP, UTP, LPS and the combination of LPS with ATP and UTP; **(C, D)** ATP, UTP, fMLP and the combination of fMLP with ATP and UTP or **(E, F)** ATP, UTP, coincubated with *L. donovani* promastigotes or co-incubated with *L. donovani* promastigotes in the presence of ATP or UTP. Production of total ROS was measured by luminol luminescence. **(B, D, F)** Representative kinetic curves of ROS production by neutrophils. **(A, C, E)** ROS production shown by calculating the area under the curve (AUC) values. Statistical analysis by ordinary one-way ANOVA with a *post hoc* Turkey’s test. Asterisks above the bars indicate significance compared to untreated cells. n=3, *p ≤ 0,05, **p ≤ 0,01, ***p ≤ 0,001, ****p ≤ 0,0001.

The production of reactive oxygen species (ROS), the oxidative burst, is one of the most important mechanisms of neutrophil granulocytes to kill phagocytosed microbes. The ability to induce the production of ROS is characteristic for many neutrophil-activating agents. Therefore, we investigated the effect of ATP and UTP on the ROS production by human neutrophils. The production of MPO-derived ROS and extracellular superoxide was assessed by the luminol-based chemiluminescence assay. PMN were incubated in medium alone as negative control or in the presence of ATP (500 µM) or UTP (500 µM). Exposure to ATP and UTP did not induce a significant level of ROS production (**Figures 2A–F**).

A characteristic feature of low-level PMN activation is the priming, i.e., the amplification of the neutrophil’s functional response to other stimuli. We have investigated whether ATP and UTP can amplify LPS- or fMLP-induced ROS production of neutrophils. Treatment with LPS (**Figures 2A, B**) and fMLP (**Figures 2C, D**) resulted in ROS production. Importantly, exposure of LPS- and fMLP-preincubated cells to ATP and UTP markedly and significantly enhanced the production of ROS (**Figures 2A–D**). These data show that ATP and UTP alone do not induce significant level of ROS production but enhance LPS- and fMLP-induced ROS production in a synergistic manner.

Since ROS production is regarded as an essential mechanism of killing of *Leishmania* by neutrophils (29) next we investigated the effect of ATP and UTP on the ROS production of neutrophils in response to *L. donovani*. Coincubation of neutrophils with *L. donovani* promastigotes induced a low and statistically not significant level of ROS production (**Figures 2E, F**). Simultaneous exposure of neutrophils to *L. donovani* and to ATP or UTP, however, led to strong ROS production (**Figures 2E, F**). These data show that ATP and UTP enhance *Leishmania*-induced ROS production in a synergistic manner.

### Phagocytic Activity of Neutrophils Is Enhanced by Exposure to ATP

**Figure 3:**
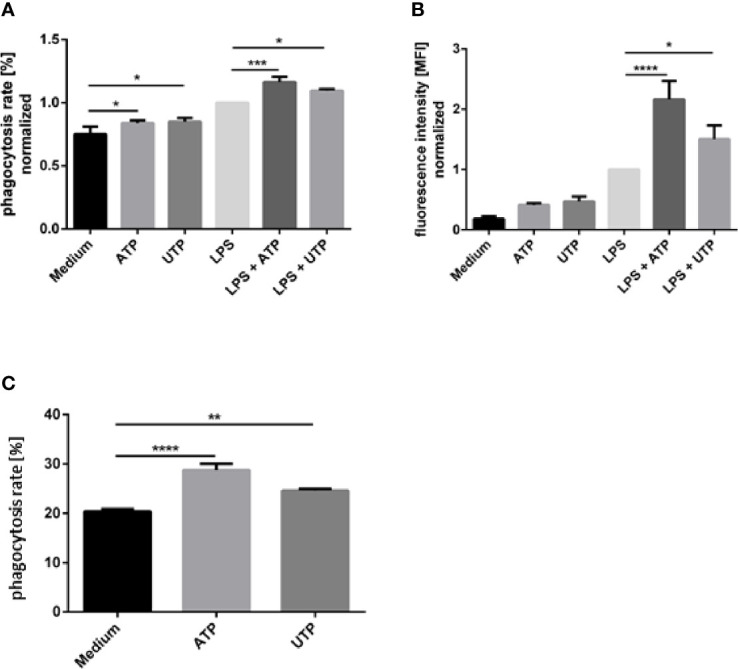
The effect of extracellular ATP and UTP on the phagocytic capacity of neutrophils. **(A, B)** Neutrophils were exposed to of ATP (500 µM) or UTP (500 µM) in the presence or absence of LPS (100 ng/ml) or left untreated. FluoSphere carboxylate-modified latex microspheres with a diameter of 1 μm were added and incubated for 30 min. Phagocytosis was assessed by flow cytometry by analyzing the ratio (%) of neutrophils with ingested fluorescent beads **(A)** and mean fluorescence intensity (MFI) of cells that phagocytosed beads **(B)**. **(C)** Neutrophils were co-incubated with CFSE-labeled *L. donovani* promastigotes for 120 min at a neutrophil to *Leishmania* ratio of 1:5. Phagocytosis was assessed by flow cytometry by analyzing the ratio (%) of neutrophils with ingested CFSE-labeled *Leishmania*. Statistical analysis by ordinary one-way ANOVA with a *post hoc* Turkey’s test. n=3, *p ≤ 0,05, **p ≤ 0,01, ***p ≤ 0,001, ****p ≤ 0,0001.

Next, we investigated whether ATP and UTP, in addition to their effects on ROS production, also activate other antimicrobial effector functions of neutrophils. Since purinergic signaling was recently shown to enhance phagocytic capacity of macrophages (30) we hypothesized that ATP and UTP can exert an activating effect on phagocytosis by neutrophils. Neutrophils were exposed to ATP and UTP and subsequently phagocytosis of fluorescence-labeled latex beads was analyzed by flow cytometry. Exposure of neutrophils to ATP and UTP only slightly enhanced the phagocytosis of latex beads by neutrophils (**Figure 3A**). Exposure to LPS enhanced both the ratio of phagocytosing cells (**Figure 3A**) and the number of phagocytosed particles per neutrophil, shown as mean fluorescence intensity (MFI) (**Figure 3B**). Simultaneous exposure of LPS-stimulated neutrophils to ATP and UTP strongly and synergistically enhanced the LPS-induced phagocytic capacity of neutrophils (**Figures 3A, B**).

Prerequisite of intracellular killing of *Leishmania* by neutrophils is the ingestion of the parasites. The effect of ATP and UTP on the ingestion of *Leishmania* parasites was assessed by using CFSE-labeled *L. donovani* promastigotes. Exposure to both ATP and UTP enhanced the phagocytosis of *Leishmania* by neutrophils (**Figure 3C**).

### Exposure to ATP and UTP Leads to Enhanced Killing of Intracellular *Leishmania* by Neutrophils

Neutrophils can play both protective as well as disease promoting roles in leishmaniasis. On the one hand neutrophils can kill intracellular *Leishmania*. On the other hand, if intracellular *Leishmania* are not killed, neutrophils can provide an intracellular niche for the survival of these intracellular pathogens. Our finding, that exposure to extracellular ATP and UTP induces ROS production (**Figure 2**) suggests that neutrophils can kill *Leishmania* at an enhanced level. The enhanced ingestion of the parasites (**Figure 3C**), on the one hand may facilitate the killing of more parasites. However, on the other hand, if the parasites are not killed, enhanced ingestion of *Leishmania* may also lead to the survival of more parasites.

**Figure 4:**
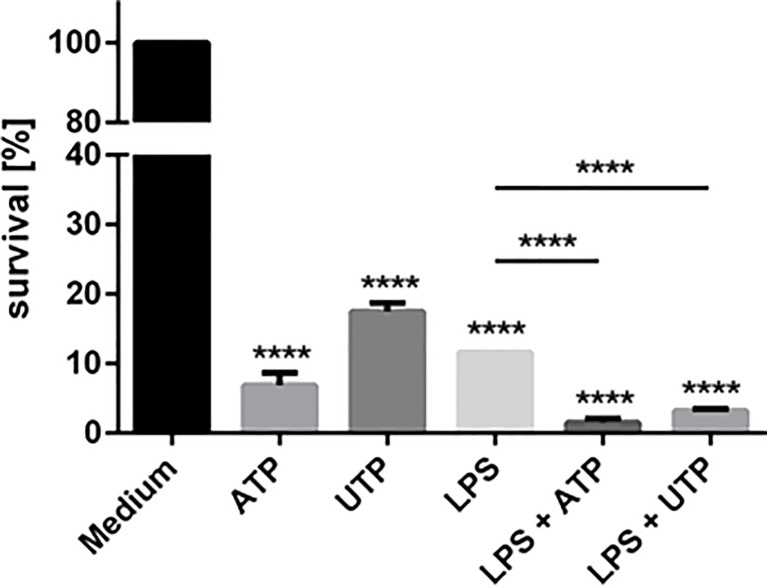
The effect of extracellular ATP and UTP on the intracellular killing of *Leishmania donovani* parasites by neutrophils. Primary human neutrophils were infected with *L. donovani* promastigotes (ratio 1:10) for 3 h at 37°C, 5% CO_2_. The infection rate was controlled on Giemsa-stained cytocentrifuge slides. After removing the free, non-ingested parasites the infected cells were treated with ATP, UTP, LPS and the combination of LPS with ATP or UTP or left untreated (medium). After 18 h of incubation at 37°C, 5% CO_2_ the survival of *L. donovani* parasites was determined by using a limiting dilution assay. The bars show % of viable *Leishmania* compared to untreated neutrophils ± SD (n=3). Statistical analysis by ordinary one-way ANOVA with a *post hoc* Turkey’s test. Asterisks above the bars indicate significance compared to untreated cells. ****p ≤ 0.0001.

To test whether exposure to ATP and/or UTP can enhance antileishmanial activity of neutrophils *Leishmania donovani*-infected neutrophils were exposed to ATP and UTP and the intracellular killing of the pathogens was assessed. Exposure of neutrophils to ATP and UTP significantly and markedly increased the killing of *L. donovani* (**Figure 4**). Exposure of *L. donovani* promastigotes to ATP and UTP did not affect the viability of the parasites indicating that ATP and UTP do not directly kill *L. donovani* promastigotes (data not shown). Therefore, the increased killing of *L. donovani* is due to activation of neutrophil anti-leishmanial activity by ATP and UTP.

As shown in **Figure 2**, ATP and UTP synergize with other activating stimuli such as LPS and fMLP. Since in preliminary studies we observed that exposure to LPS leads to enhanced anti-laishmanial activity of neutrophils (data not shown) we investigated whether the ATP and UTP exert a synergistic effect with LPS regarding the capacity of neutrophils to kill intracellular *Leishmania*. Simultaneous exposure of *Leishmania*-infected neutrophils to LPS and ATP or UTP enhanced significantly the killing of the parasites in a synergistic manner (**Figure 4**).

### Exposure to ATP and UTP Results in Enhanced Production of Pro-Inflammatory Cytokines by Neutrophils

In addition to their antimicrobial effector functions neutrophils can regulate inflammatory and immune responses by releasing cytokines (31). After having seen that extracellular ATP and UTP can activate effector functions of neutrophils we investigated whether exposure to ATP and UTP can also influence the proinflammatory regulatory functions of neutrophils. Such a function can contribute to the antimicrobial effects of ATP and UTP *in vivo*.

**Figure 5:**
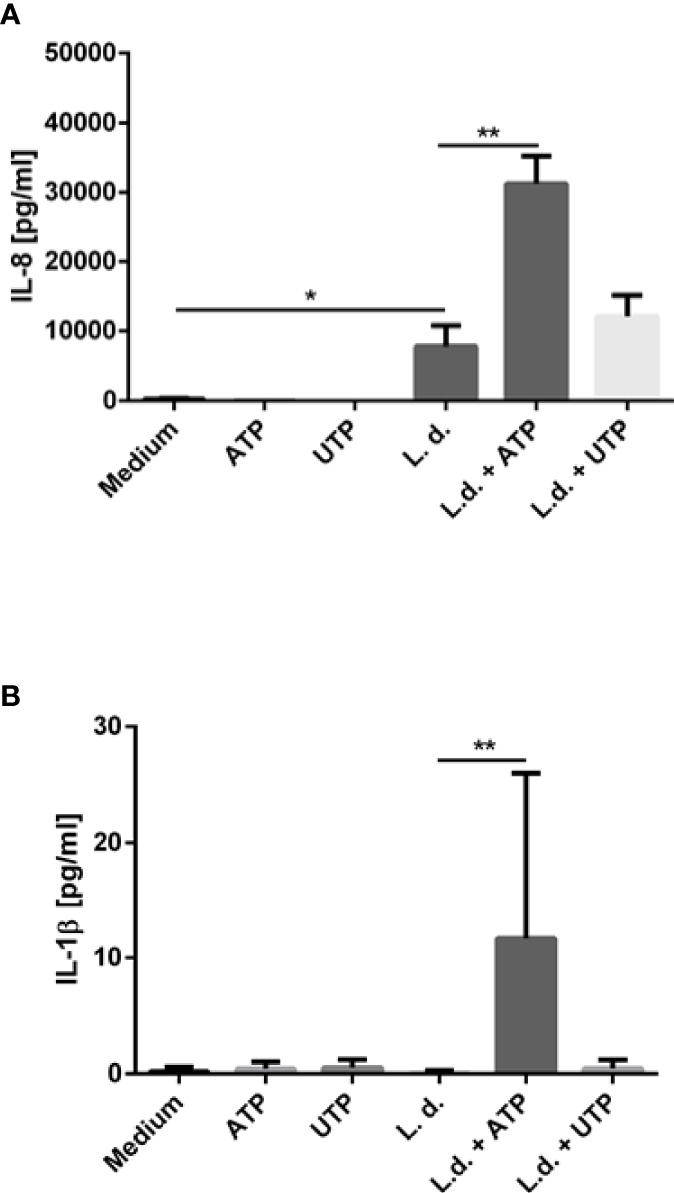
The effect of extracellular ATP and UTP on the *Leishmania*-induced cytokine production by neutrophils. Primary human neutrophils were infected with *L. donovani* promastigotes (ratio 1:10) for 3 h at 37°C, 5% CO_2_. After removing the free, non-ingested parasites the infected cells were treated with ATP, UTP or left untreated for 18 h. The IL-8 content **(A)** and the IL-1β **(B)** content of the supernatants was analyzed by ELISA. n=3, *p ≤ 0,05, **p ≤ 0,01.

IL-8 (CXCL8) is a major chemotactic factor for neutrophils. However, neutrophils not only respond to but also secrete IL-8 (32). This autocrine mechanism is thought to serve as positive feedback for neutrophil recruitment. Because neutrophil recruitment is generally considered to be proinflammatory, we tested the effect of extracellular ATP and UTP on the IL-8 release by human neutrophils. Neutrophils were exposed to *L. donovani*, ATP or UTP or to the combination of *L. donovani* with ATP or UTP for 18 h and the IL-8 content of the supernatants was analyzed by ELISA. Exposure to ATP or UTP alone did not induce the release of IL-8 (**Figure 5A**). Co-incubation with *L. donovani* promastigotes resulted in the release of high IL-8 levels (**Figure 5A**). Importantly, extracellular ATP strongly enhanced the *L. donovani*-induced IL-8 release (**Figure 5A**). Exposure to UTP enhanced slightly the *Leishmania*-induced IL-8 release. However, this effect was not statistically significant (**Figure 5A**).

Activation of purinergic receptors plays also a major role in the immune responses through the secretion of pro-inflammatory cytokines. Extracellular ATP was reported to activate the NALP3 inflammasome which initiates a cascade of proteolytic events commencing with the conversion of procaspase-1 to caspase-1 resulting in the processing and secretion of IL-1β (33). In our present study co-incubation of neutrophils with *L. donovani* did not induce the release of IL-1β (**Figure 5B**). Exposure to extracellular ATP or UTP also did not induce IL-1β release (**Figure 5B**). Simultaneous exposure to *L. donovani* and ATP, however, resulted in the release of IL-1β from primary human neutrophils (**Figure 5B**).

### Exposure to ATP and UTP Results in Enhanced Intracellular Ca^2+^-Concentration

**Figure 6:**
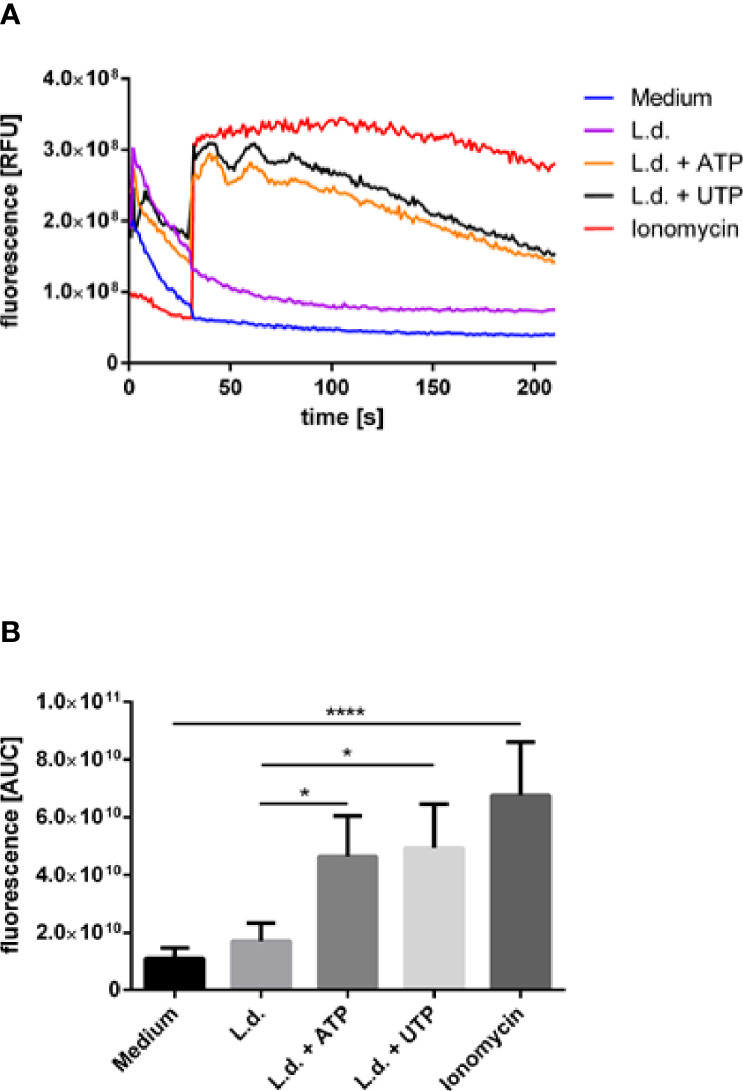
The effect of extracellular ATP and UTP on the intracellular Ca^2+^-concentration in *L. donovani*-infected neutrophils. Neutrophils were labeled with the fluorescent Ca^2+^-sensitive indicator Fluo-4 AM and infected with *L. donovani* promastigotes. Infected neutrophils were exposed to ATP (500 µM) or UTP (500 µM) and the fluorescence intensity was assessed for 3 min by using flow cytometry. The stimuli were added at the time point 30 s of the flow cytometry analysis. Fluo-4-labeled non-infected cells were used as negative controls, Fluo-4-labeled non-infected cells exposed to ionomycin (10 µM) were used as positive controls. **(A)** Representative kinetic curves of Fluo-4 AM fluorescence in neutrophils. **(B)** Fluo-4 AM fluorescence shown by calculating the area under the curve (AUC) values calculated between the time points 30 sec and 210 sec. Statistical analysis by ordinary one-way ANOVA with a *post hoc* Turkey’s test. Asterisks above the bars indicate significance compared to untreated cells. n=3, *p ≤ 0,05, ****p ≤ 0,0001.

Purinergic signaling has been described to lead to Gα_q_-dependent activation of phospholipase C-β (PLCβ) resulting in the hydrolysis of PtdIns (4,5)P_2_ producing the second messengers Ins (1,4,5)P_3_ which then in turn mobilized intracellular Ca^2+^ (30). In order to investigate whether this pathway can be involved in the enhanced killing of *Leishmania* in neutrophils upon exposure to ATP and UTP we next assessed the effect of extracellular ATP and UTP on Ca^2+^ mobilization in *Leishmania*-infected neutrophils. A strong intracellular Ca^2+^ mobilization was observed in infected neutrophils upon treatment with ATP and UTP (**Figure 6**). Treatment with ionomycin was used in these experiments as positive control (**Figure 6**).

### Extracellular ATP Induces Phosphorylation of Akt, Erk1/2 and p38 MAPK in *L. donovani*-Infected Neutrophils

**Figure 7:**
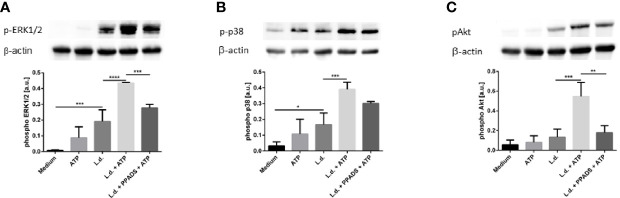
The effect of extracellular ATP and UTP on the phosphorylation of p38, Akt and ERK1/2 in neutrophils. Neutrophils were exposed to ATP (500 µM), UTP (500 µM), *Leishmania donovani* promastigotes (*Leishmania* to neutrophil ratio 10:1) or a combination of ATP or UTP with *Leishmania donovani* promastigotes for 15 min at 37°C. In samples with the P2-antagonist PPADS the neutrophils were pretreated with the inhibitor (30 µM) for 15 min. Whole cell lysates were prepared and phosphorylation of ERK1/2 **(A)**, p38 **(B)** and Akt **(C)** were analyzed by Western Blot. Representative blots are shown in the upper parts of the panels. Phosphorylation of ERK1/2, p38 and Akt was quantified by densitometry analysis. Signals of pAkt, pERK1/2, or pp38 were related to β-actin signals on the same blots. n=4, *p ≤ 0,05, **p ≤ 0,01, ***p ≤ 0,001, ****p ≤ 0,0001.

Purinergic signaling has been described to lead to Gα_q_-dependent activation of phospholipase C-β (PLCβ). Activation of PLCβ results in the hydrolysis of diacylglycerol (DAG) which activates protein kinase C (PKC) which then in turn activates the serine/threonine kinase ERK1/2 (34). Signaling events involving Akt, Erk1/2 and p38 MAPK are also involved in inflammasome activation and the subsequent processing of IL-1β. Having observed a synergistic effect of ATP and *L. donovani* on the IL-1β-release of human primary neutrophils (**Figure 5**) we next investigated whether phosphorylation of Akt, Erk1/2 and p38 MAPK is affected upon simultaneous contact of neutrophils to *L. donovani* and ATP. Western blot analysis reveled an upregulation of the phosphorylation of Akt, Erk1/2 and p38 MAPK upon exposure to extracellular ATP in *L. donovani*-infected neutrophils (**Figure 7**).

In order to demonstrate that the observed phosphorylation events are induced by signaling *via* purinergic receptors the effect of the non-selective P2 purinergic receptor antagonist PPADS was investigated. Treatment with PPADS strongly and significantly reduced the phosphorylation of ERK1/2 and Akt in neutrophils exposed to *L. donovani* and ATP (**Figures 7A, C**). The inhibitory effect of PPADS on the phosphorylation of p38 MAPK was apparent, however, statistically not significant (**Figure 7B**).

### Exposure to Extracellular ATP and UTP Does Not Compromise Viability of Neutrophils

Since in our experiments a relatively high concentration (500 µM) of ATP and UTP was applied to neutrophils for a time period varying between 15 min and over night exposure, we tested the effect of ATP and UTP treatment on the apoptosis and viability of neutrophils. Neutrophils are short lived cells, they undergo spontaneous apoptosis within hours both *in vivo* and *in vitro* (35, 36). Apoptosis and viability of neutrophils were assessed after 1h, 4h and 20h exposure to 500 µM of ATP and UTP. Extracellular ATP and UTP neither enhanced apoptosis nor exerted any toxic effects (**Supplemental Figure 1**). In contrary, exposure to ATP and UTP showed a tendency to reduce apoptosis and enhance viability. This effect was statistically significant after 20h exposure of neutrophils to ATP (**Supplemental Figure 1**).

## Discussion

In the present study we demonstrated that extracellular ATP and UTP results in enhanced anti-leishmanial effector functions of neutrophil granulocytes *in vitro*. We could show that activation status, granule degranulation, ROS-production, phagocytic capacity of primary human neutrophils and intracellular Ca^2+^ concentration are enhanced upon exposure to ATP and UTP. Exposure of *Leishmania*-infected neutrophils to ATP and UTP resulted in a strongly enhanced ability of neutrophils to kill the intracellular parasites. Importantly, exposure to ATP resulted in the production and release of strongly enhanced levels of IL-8 and IL-1β by *Leishmania*-infected neutrophils. We could show that simultaneous exposure to *Leishmania donovani* promastigotes and ATP leads to the phosphorylation of ERK1/2, p38 MAPK and Akt and that P2 purinergic receptors are involved in these phosphorylation events.

At inflammatory foci the concentration of ATP and other purine and pyrimidine nucleotides can be very high, the ATP concentration was shown to reach hundreds of micromolar concentrations (11). These high concentrations have significant biological effects. *In vitro* studies with ATP showed significant biological effect of extracellular ATP in the concentration of 10^-3^ -10 ^-4^ M (37). ATP (10^-3^ -10 ^-4^ M) also leads to mobilization of intracellular Ca^2+^ (38). In an *in vitro* study ATP exerted an inhibitory effect on spontaneous neutrophil apoptosis in concentrations between 100 and 1000 µM (39). Based on these findings in our present study we used ATP and UTP at a concentration of 500 µM. In our study, exposure to ATP and UTP also showed a tendency to reduce apoptosis and enhance viability. This effect was statistically significant after 20h exposure of neutrophils to ATP.

Purinergic activation of macrophages by ATP and UTP was shown reduced parasite load in *Leishmania* infected macrophages (15). Parasite load in *L. amazonensis* infected macrophages was reduced *in vitro* after treatment with UTP (17). Enhanced elimination of intracellular *Leishmania* by macrophages has been described upon exposure to extracellular ATP (40) and UTP (41). By using the air pouch technique UTP treatment of *Leishmania*-infected mice was shown to induce neutrophil recruitment and enhanced ROS production. However, the source of ROS was not investigated in this study. We hypothesized that neutrophils were the likely source of ROS after UTP treatment. Since neutrophils were recently recognized as host cells for the intracellular parasite *Leishmania* our present study focused on the effect of extracellular ATP on UTP on the functions and, importantly, on the capacity of neutrophils to kill intracellular *Leishmania*.

Previous studies described the activating effect of purinergic regulation on selected individual function of neutrophils such as degranulation (37, 42), ROS production (42) and mobilization of intracellular Ca^2+^ (38) we here followed a more comprehensive approach to investigate neutrophil functions and investigate these functions in relation to infection with *Leishmania donovani*.

The production of reactive oxygen species (ROS) is a major antimicrobial effector functions of neutrophils. This function is regarded as essential mechanism to kill intracellular *Leishmania*. We observed that ATP or UTP alone did not induce the production of significant levels of ROS. Exposure to *Leishmania* also did not induce the production of significant ROS levels. Importantly, in the presence of ATP and UTP high ROS levels were produced upon exposure to *Leishmania* parasites. We observed similar synergistic effects of ATP and UTP with the microbial stimuli LPS and fMLP. Purinergic activation was shown to be closely connected to other activating signaling pathways. Extracellular ATP was shown to synergize with TNF-alpha for the activation of DCs (43). A good example for the connection between purinergic and other signaling pathways is our finding regarding the secretion of IL-1β upon exposure of *Leishmania*-infected neutrophils to ATP. It is generally accepted that the activation of NLRP3 inflammasome require two signals, *i*) a priming signal which results in the upregulation of NLRP3 and pro-IL-1β and *ii*) and a second signal leading to the assembly of the NLRP3 inflammasome complex (44). The first signal is provided by pattern recognition receptors recognizing microbial constituents such as toll like receptors (TLR) (45). The second signals include DAMPs such as extracellular nucleotides (46). Accordingly, ATP-induced release of IL-1β was shown to require a priming step by LPS (47).

Several pattern recognition receptors have been reported to be involved in the recognition of *Leishmania* parasites such as TLR2 (48), TLR4 (49), TLR9 (50) and the NLR (reviewed in 51). Importantly, these receptors are known to provide a signal for NLRP3 activation. NLRP3-IL-1β axis is important for the defense against *Leishmania amazonensis*. *Leishmania*-induced activation of NLRP3 inflammasome and subsequent IL-1β production facilitated host resistance to *Leishmania* infection in macrophages *in vitro* and in mice *in vivo* (52). Extracellular ATP at a concentration of 500 µM was reported to induce IL-1β signaling and control of *L. amazonensis* infection in macrophages *in vitro* (53). Extracellular ATP was shown to activate NLRP3 inflammasome in murine neutrophils resulting in IL-1β secretion (34). Here we showed that extracellular ATP acts as second signal in *Leishmania*-infected human neutrophils to induce IL-1β release.

Extracellular ATP was shown to be involved in the stimulation of neutrophils with fMLP but also through receptors for IL-8, C5a and LTB4 (20). The production of LTB4 has been reported to be involved in the ATP- and UTP-mediated killing of *Leishmania* by macrophages (15, 54). In a previous study we demonstrated that neutrophils infected with *Leishmania* release an increased amount of LTB4 (55). It is, therefore, tempting to hypothesize that neutrophil-derived LTB4, in an autocrine manner, is involved in the ATP- and UTP-mediated enhanced elimination of intracellular *Leishmania* by neutrophils.

ATP and other nucleotides such as UTP are released from cells at sites of inflammation in high concentrations. Extracellular ATP and related purine and pyrimidine nucleotides exert their functions as danger signals *via* signaling through membrane-bound purinergic P2 receptors. P2 receptor signaling is mediated by pathways involving the phosphorylation of ERK1/2, Akt and p38 MAPK (34). Having seen the activating effects of extracellular nucleotides on neutrophil function we used an inhibitor approach to show that the observed effects were mediated by purinergic receptor(s). Extracellular ATP and UTP act on leukocytes upon binding to surface P2 receptors. ATP is an agonist for all seven P2X receptors and can bind to all eight P2Y receptors except to the P2Y6 receptor (12–14). UTP is an agonist of P2Y2, P2Y4 and P2Y11 (12, 13). To obtain a hint regarding the receptors involved in the observed affects the non-selective P2 receptors antagonist PPADS was used. By using PPADS we could show the involvement of P2-receptor(s) in the ATP-induced signaling events that involve the phosphorylation of ERK1/2, Akt and p38 MAPK.

Taking together our results show that extracellular ATP and UTP activate anti-leishmanial effector and also regulatory functions of human neutrophils. Therefore, therapy options using extracellular nucleotides can target *Leishmania*-infected neutrophils leading to enhanced elimination of parasite load. Moreover, since the presence of high numbers of neutrophils and high extracellular ATP and UTP-concentrations is characteristic for multiple inflammatory and infectious conditions, our results suggest that ATP- and UTP-induced activation of neutrophils has a major and clinically relevant pathophysiological impact. In one hand neutrophils activated by extracellular ATP and/or UTP exert an enhanced antimicrobial activity. On the other hand neutrophils activated by extracellular ATP and/or UTP may have the potential to exert a disease promoting effect in chronic or autoinflammatory conditions.

## Data Availability Statement

The raw data supporting the conclusions of this article will be made available by the authors, without undue reservation.

## Ethics Statement

The studies involving human participants were reviewed and approved by Ethical Committee of the Medical Faculty of the University of Lübeck. The patients/participants provided their written informed consent to participate in this study.

## Author Contributions

SM conducted laboratory work, analysed data, and prepared the figures. TL designed the study, analysed data, and wrote the manuscript. All authors contributed to the article and approved the submitted version.

## Conflict of Interest

The authors declare that the research was conducted in the absence of any commercial or financial relationships that could be construed as a potential conflict of interest.

## Publisher’s Note

All claims expressed in this article are solely those of the authors and do not necessarily represent those of their affiliated organizations, or those of the publisher, the editors and the reviewers. Any product that may be evaluated in this article, or claim that may be made by its manufacturer, is not guaranteed or endorsed by the publisher.
