# Supplementary material for: Purinergic Enhancement of Anti-Leishmanial Effector Functions of Neutrophil Granulocytes
Source: Front Immunol. 2021 Oct 18;12:747049. doi: 10.3389/fimmu.2021.747049 (PMC8558537; doi:10.3389/fimmu.2021.747049)
Supplement: Supplementary file 1 [file Image_1.pdf]

Supplemental Figure 1

A

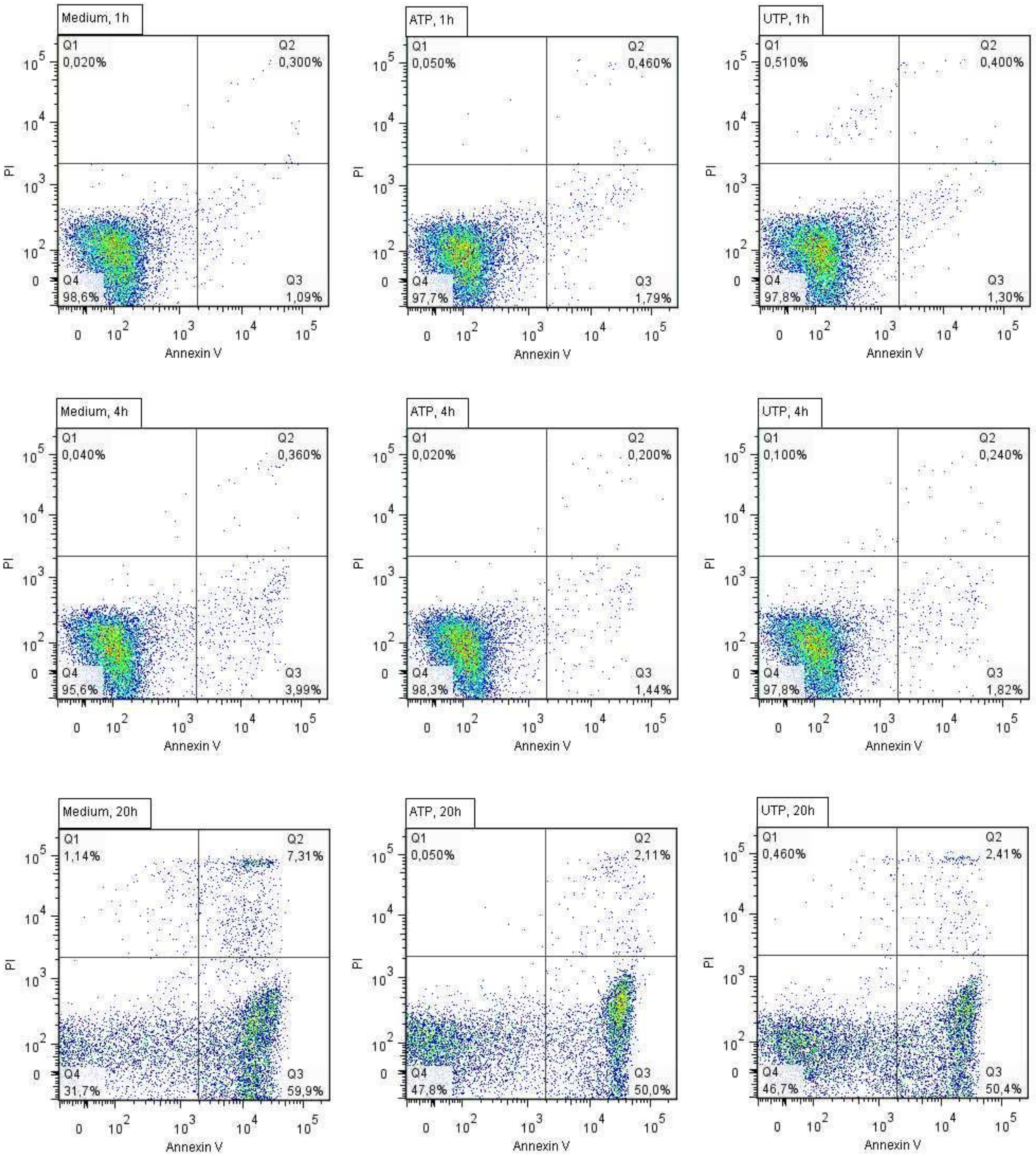

B

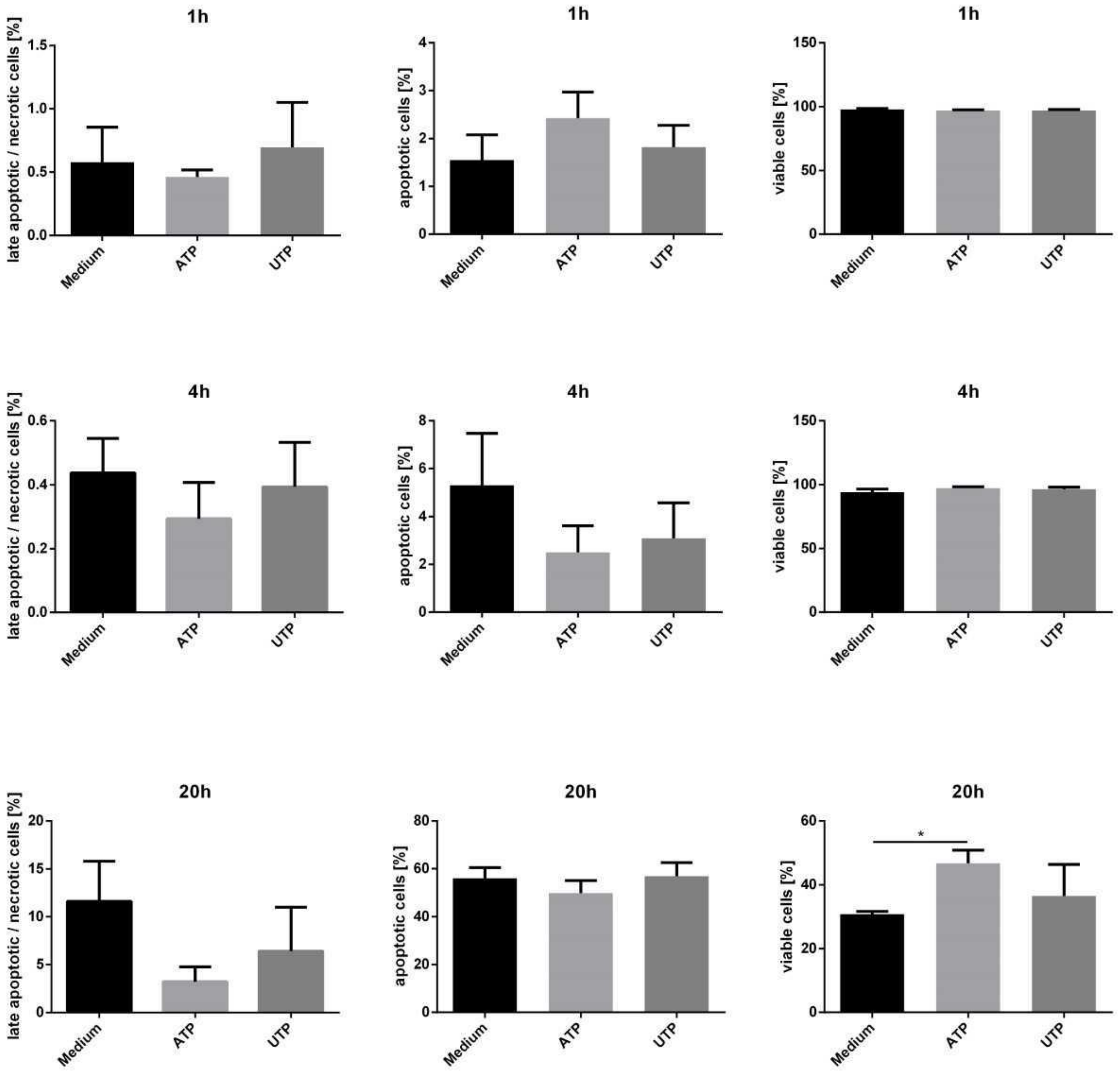

Supplemental Figure S1
The effect of extracellular ATP and UTP on the apoptosis and viability of primary human neutrophils.

Primary human neutrophils were incubated for 1h, 4 h and 20h in the presence of ATP (500 μM) or UTP (500 μM). Apoptosis and necrosis of neutrophils was determined by staining with Annexin-V FLUOS and propidium iodide (PI), respectively, and analyzed by flow cytometry.
A) Representative dot plots. Double negative cells (Q1) represent viable cells. Annexin-V-positive PI-negative cells (Q4) represent apoptotic cells. Double positive cells (Q2) represent late apoptotic/necrotic cells. **B)** Bar diagrams showing the ratio of viable, apoptotic and late apoptotic/necrotic cells. n=3, *=p≤0,05
